# Surgical Importance of the Caput Coli, and Its Etiologic Factorate in Producing Inflammation of the Vermiform Appendix1Read at the 35th annual meeting of the Medical Association of Central New York, held at Syracuse, N. Y., October 21, 1902.

**Published:** 1902-12

**Authors:** J. P. Creveling

**Affiliations:** Auburn, N.Y., 22 South Street.


### Surgical Importance of the Caput Coli, and Its Etiologic Factorate in Producing Inflammation of the Vermiform Appendix.[1]

By J. P. CREVELING, M.D., Auburn, N. Y.

IN OFFERING a few remarks upon a subject of which the foregoing is a sufficiently descriptive title, I wish to invite attention to the fact that anatomically the cecum is the receptacle for all material passing through the alimentary canal. It is the only blind pouch into which that material normally goes, and it is at a great disadvantage to discharge its contents. During the upright position it necessarily becomes a pocket for the digestive tract, with the force of gravity to overcome. The

1. Read at the 35th annual meeting of the Medical Association of Central New York, held at Syracuse, N. Y., October 21, 1902.

ileocecal valve prevents the crowding of the contents back into the small intestines and the ascending colon, running vertically as it does, throws on the cecum a greater effort to clear its cavity than any other part of the tract.

Being thus located at the base of a practically vertical tube, the position renders it particularly liable to disease, especially of an infectious and inflammatory nature. The latter process occurring here with the deposition of plastic material may so hamper the action of the bowel that fecal matter is retained within the distorted lumen, and it becomes a nest of infection, particularly for the appendix, the orifice of which is only incompletely guarded and, with structural changes interfering with the muscular closure, it is subjected to increased risk not only of infection, but to foreign substances as well.

We have all seen this organ filled with refuse material before other evidence of disease. This is most likely to occur when the cecal pouch is crippled by inflammatory adhesions. From simularity of structure and position a morbid process in one is prone to extend to, and involve the other, or a cecitis may often mean a subsequent appendicitis, or the converse may take place. The symptoms, clinical manifestations and structural changes in the two are in no way dissimilar, except as influenced by the size of the canal.

Writers of recent years, both medical and surgical, seem to ignore the pathologic importance of this bowel, but Flint in his theory and practice of 1866, gives a brief but lucid exposé of the symptoms and changes that are present in inflammatory conditions of this part. After speaking of the lesions, he says the pain and tenderness are in the region of the cecum with vomiting, diarrhea and febrile movement, and adds that this condition is liable to be confounded with local peritonitis and perforation of the vermiform appendix.

In referring to the latter disease, he gives a succinct clinical description, and then practically locates the now McBurney point in the following words, "the focus of tenderness being on a horizontal line from the crest of one ilium to the other, and just external to the right rectus muscle." He ascribed the general symptoms and peritonitis to local lesions of these parts, but it took some time for the surgical element of the profession to turn these observations to practical account.

A somewhat careful study of diseases occurring in the ileocecal fossa, that have come under my observation, has led me to believe that a large percentage of the disorders involving the colon, appendix and adjacent structures have their origin in the cecum, and in many instances are mere extensions from continuity of surface. After extensive morbid changes and pus accumulation it is not always easy to determine in what part the disease began. To me it is quite impossible to differentiate the two conditions when certain symptoms prevail and some of us have opened the abdomen for appendicular trouble and found that organ involved secondarily, or even in a comparatively healthy state.

A very marked instance in this direction occurred with me some weeks ago. I was called to a neighboring town to operate for acute appendicitis, and on the way from the train to the house of the patient, the attending physician said, "this is the most typical case I have ever seen, every symptom is just right and characteristic. The pain, tenderness, muscular rigidity, vomiting and all other signs are in proper form." I looked the case over with considerable care and was obliged to confirm his diagnosis. An operation was advised and immediately performed, but when the abdomen was opened, the appendix was found to be small and quite normal, except at the cecal end for half an inch or so down the tube.

The cecum, however, was much inflamed and already well covered by inflammatory product which had fastened it to the parietal wall. There could be no doubt but that the disease in the vermiform extension was secondary to and due to progressive inflammation in the cecum. The appendix was removed and carefully examined, but seemed to be in a normal condition, except as stated, and the serous coat was principally involved; the muscular wall and the mucous coverings showing scarcely a trace of disease.

The point at issue is, the cecum was liberated from the pent down position, adhesions broken up, and the bowel returned to the cavity free. On the day of the operation the temperature was 102°, pulse, 98; but on the following day both had receded to normal. Now there is no doubt that the opening of the abdomen and treatment of the cecum relieved the inflammatory process then going on, and the bowel returned to its normal condition, capable of performing its functions.

I wish very briefly to give the outlines of another case that likewise illustrates not only the difficulty in differentiation but also the more important point, the benefit derived from operation. Seven years ago I attended a girl then 14 years old through an attack of what presented all the indications of a very acute appendicitis. It was a severe seizure with all symptoms highly pronounced. The pain, tenderness, vomiting, muscular rigidity, and on the tenth day an oval tumor appeared in the proper place. No surgical treatment could be adopted and it required two months for her to recover. From that time until January, 1902, she had had a number of milder attacks, lasting only a few days and then passing away. I had regarded the case as one of recurrent appendicitis and that opinion had been indorsed by a number of men of experience, as some of the attacks occurred in other cities and at summering places. In January last she was again taken with pain and tenderness as before, and by rest, diet and treatment for two weeks the acuteness was overcome, but soreness remained, and except when absolutely quiet she had pain. In February last the abdomen was opened, and as the peritoneum was divided it was seen that the cecum was adherent to the anterior wall of the abdomen to near its entire length. It was dissected loose and brought well out of the wound and on the dorsum lay the little appendix, rather under size, without any appearance of disease or indications of ever having been diseased. She made a good recovery, and has since been entirely free from disturbance in this region. Here, again, there is no doubt as to the lesion causing the attacks, or that the surgical intervention so relieved the condition that further trouble has not to this date reappeared. The tumor during the first attack, I presume, was due to the local peritonitis as a result of the cecitis.

These two are not entirely isolated cases, as others of a similar character might be cited, but they stand as exponents of the position taken, that much of the disease in this region of the abdomen is due to disorder of the cecal tract, and also, that much of such condition is amenable to surgical treatment. The colicky pains in this locality met with in some cases, occurring at intervals for months and even years, without getting materially worse or better, and looked upon as recurrent appendicitis are in some instances undoubtedly due to cecal adhesions. Such adhesions interfere with the full physiologic function of the bowel and may be relieved by operative measures. Another pathologic condition that may take place here, and of which I have seen but one case, is perforation of the intestine.

In this instance a fecal accumulation much too large to be retained in the appendix, was found floating in the cavity of the abscess. On investigation it was seen that the bolus had escaped through the end of the cecum by way of ulceration. In this case the caliber of the bowel was constricted by inflammatory exudate and the pouch extremely distended. The necrotic condition was probably due to pressure from within, and this in turn to the constriction. The large size of the intestine usually prevents complete occlusion, but the narrowing of the lumen or adhesions to the abdominal wall interferes with normal action, and either is liable to give rise to all the symptoms enumerated. Both conditions are relieved by operative measures and should be a part of surgery.

22 SOUTH STREET.

